# Phenotypic and genotypic characterization of macrolide resistance among *Staphylococcus aureus* isolates in Isfahan, Iran

**Published:** 2017-10

**Authors:** Hossein Sedaghat, Bahram Nasr Esfahani, Sina Mobasherizadeh, Azhar Sallari Jazi, Mehrdad Halaji, Parisa Sadeghi, Mohammad Emaneini, Seyed Asghar Havaei

**Affiliations:** 1 Department of Microbiology, School of Medicine, Isfahan University of Medical Sciences, Isfahan, Iran; 2 Nosocomial Infection Research Center, Isfahan University of Medical Sciences, Isfahan, Iran; 3 Department of Microbiology, School of Medicine, Tehran University of Medical Sciences, Tehran, Iran

**Keywords:** *Staphylococcus aureus*, Inducible resistance, D-test, *erm* A, *erm*C, *msr*A

## Abstract

**Background and Objectives:**

Macrolide, lincosamide and streptogramin B (MLS _B_) are noteworthy antibiotics for the treatment of *Staphylococcus aureus* infections. The purpose of this study, was to determine the phenotypic and genotypic characterization of macrolide resistance, among *S. aureus*, isolated from clinical samples and nasal swabs.

**Materials and Methods:**

Totally, 162 non-duplicate *S. aureus* isolates were collected from clinical samples and nasal swabs, from patients and healthcare workers (HCWs), between March 2016 and September 2016, at four teaching hospitals in Isfahan. The antibiotic resistance profile was determined using disk diffusion test and the presence of resistance genes was detected, using PCR.

**Results:**

Of 162 *S. aureus* isolates, 43.8% (71/162) and 34% (55/162) isolates were erythromycin-resistant and methicillin-resistant *S. aureus* (MRSA), respectively. The prevalence of constitutive MLS _B_ (cMLS _B_), inducible MLS _B_ (iMLS _B_), macrolide-streptogramin B-resistant (MS _B_) and lincosamide-streptogramin-A resistance (LS _A_) phenotype was 32%, 6%, 6% and 2%, respectively. The most common erythromycin resistance genes, in *S. aureus* isolates were *erm*C (35.2%), followed by *erm*A (20.4%) and *msr*A (17.3%). Meanwhile, *msr*A was detected in 43.6% of MRSA isolates. The frequency of coexistence of *erm*A+*erm*C+*msr*A, in *S. aureus* isolates was 7% and it was only detected in MRSA isolates.

**Conclusion:**

In the current study, cMLS _B_ phenotype was the most common erythromycin resistance pattern and *erm*C was the most prevalent gene in erythromycin-resistant isolates. The results revealed that the various mechanisms of erythromycin resistance are expanding in Isfahan.

## INTRODUCTION

Macrolide, lincosamide and streptogramin type B (MLS _B_) are antibiotics with different chemical structures, but with the same activity. The mechanism of action of these antibiotics is to bind to 23s rRNA in 50s ribosomal subunits, and subsequently inhibiting the protein synthesis. These antibiotics are used in the treatment of a wide range of bacterial infections (1–3). These drugs are particularly used, in the treatment of staphylococcal infections, including skin and soft tissue infections (4, 5). The emergence of multi-drug resistant *S. aureus* strains, such as methicillin-resistant *S. aureus* (MRSA), has become a health concern due to the limited treatment options. Furthermore, *S. aureus* nasal colonization has increased the concerns about the transmission of these strains and development of different types of infections, with resistance to these noteworthy antibiotics (6, 7).

The most common mechanism of macrolide resistance is methylation of the ribosomal target of the antibiotics, encoded by a variety of erythromycin ribosomal methylase (*erm*) genes among which, *erm*A and *erm*C are predominant genes in staphylococci, which can be constitutively expressed or expressed by induction (1, 6, 8). The second mechanism is drug efflux, typically mediated by the ATP-binding cassette encoded by *msr*A (1, 8, 9). It is important to identify the phenotypic patterns of MLS _B_ resistance, for establishing appropriate therapy (10). There are four different classical phenotypic patterns of MLS _B_ resistance *in vitro*. The constitutive resistance phenotype (cMLS _B_), determined by resistance to erythromycin (a macrolide) and clindamycin (a lincosamide). The inducible resistance phenotype (iMLS _B_), determined by resistance to erythromycin and flattening of the susceptible zone of inhibition to clindamycin, adjacent to the erythromycin disk (D-shape) in D-test. The MS _B_ phenotype determined by resistance to erythromycin disk and susceptibility to clindamycin disk in D-test, and the LS _A_ phenotype determined by susceptibility to erythromycin disk and resistance to clindamycin (11). cMLS _B_ strains are resistant to all macrolides, lincosamides, and type B streptogramins, *in vivo.* Staphylococcal isolates that display iMLS _B_ phenotype should not be treated with clindamycin. Strains with iMLS _B_ phenotype may be falsely detected, as resistant to erythromycin and sensitive to clindamycin and it is important to determine iMLS _B_ phenotype by D-test, *in vitro* (12, 13).

Despite, the importance of knowing the type of MLSB resistance in a healthcare setting, no sufficient data has been focused on MLSB prevalence, among healthcare workers (HCWs) and hospitalized patients; therefore, the present study was undertaken to determine the prevalence of cMLS _B_, iMLS _B_, MS _B_ and LS _A_ resistance phenotypes and the presence of the *erm* and *msr*, in the clinical and nasal isolates of *S. aureus*, among HCWs and patients in four hospitals in Isfahan, Iran.

## MATERIALS AND METHODS

### Study design and setting.

A cross-sectional study conducted, between March 2016 and September 2016, among non-duplicate *S. aureus* isolates, obtained from clinical isolates and nasal swabs of hospitalized patients and HCWs in four teaching hospitals (Alzahra, Shariati, Imam Kazem and Shahid Chamran) in Isfahan, Iran. Samples were collected from the different wards including surgery, intensive care units (ICUs) and internal medicine. Clinical isolates were obtained from the wound, blood, urine culture, sputum, peritoneum and synovial samples. This study was in accordance with the declaration of Helsinki and informed written consent obtained from hospitalized patients and HCWs.

### Bacterial isolation and identification.

The clinical specimens were collected from hospitalized patients and transported to the laboratory for identification. For the preparation of the nasal samples a sterile swab soaked with saline was rotated in the anterior 1.5 cm of the nasal vestibule of both of the personnel and patient’s nares and subsequently inoculated into mannitol salt agar medium. After incubation at 35°C overnight, the isolates were identified as *S. aureus*, based on colony morphology, Gram staining, catalase test, coagulase test, mannitol fermentation and DNase test (14).

### Antimicrobial susceptibility testing.

Antibiotic resistance pattern was determined according to the Clinical and Laboratory Standards Institute (CLSI) guideline (15). For this purpose, a Mueller–Hinton agar plate was inoculated with suspensions of bacteria, equivalent to standard 0.5 McFarland. Subsequently, a disk of clindamycin (2 μg), was placed on media near a disk of erythromycin (15 μg), at a distance of 15–26 mm (edge to edge), and incubated at 35°C for 16–18 h. Isolates without inhibition zone around the two indicated disks were recognized as the cMLS _B_ phenotype. The iMLS _B_ phenotype was recognized based on flattening of the inhibition zone around the clindamycin disk near to erythromycin disk (positive D test). Whereas, the MS _B_ phenotype was determined by a circular zone, around clindamycin, and bacterial growth around the erythromycin disk. The LS _A_ phenotype was identified by resistance to clindamycin only, and susceptibility to erythromycin. The MRSA isolates were screened based on susceptibility to cefoxitin (30 μg) and confirmed by molecular detection of *mec*A.

### Detection of *erm*A, *erm*C, *msr*A and *mec*A genes.

DNA was extracted from *S. aureus* isolates as instructed by by Ito et al. (16). Detection of *erm*A, *erm*C, *msr*A and *mec*A was carried out with the primer sequences listed in Table 1. Amplification of genes was performed in a final volume of 25 μl containing 1μl of each primer (10 pmol), 1X PCR buffer, MgCl _2_, 0.2 mMdNTP Mix, 5 μl of template DNA and 1.5U of Taq DNA polymerase. PCR conditions were as follows: 30 cycles of denaturation at 94°C for 30s, annealing at 52° for 1min and extension at 72°C for 1 min for *erm* and 25 cycles of denaturation at 94°C for 1 min, annealing at 50°C for 1 min and extension at 72°C for 90 s for *msr*A (4). PCR conditions for detection of *mec*A were as follows: 30 cycles of denaturation (94°C, 2 min), annealing (57°C, 1 min), extension (72°C, 2 min), and a final elongation at 72°C for 2 min.

**Table 1.** Primers used in this study

**Genes**	**Sequence (5'–3')**	**Product size (bp)**	**References**
***erm*C**	F: 5'-GCTAATATTG TTTAAATCGT CAATTCC-3'	572	(34)
	R: 5'-GGATCAGGAA AAGGACATTT TAC-3'		
***erm*A**	F: 5'-GTTCAAGAAC AATCAATACA GAG-3'	421	(34)
	R: 5'-GGATCAGGAA AAGGACATTT TAC-3'		
***msr*A**	F: 5'-GGCACAATAA GAGTGTTTAA AGG-3'	940	(34)
	R: 5'-AAGTTATATC ATGAATAGAT TGTCCTGTT-3'		
***mec*A**	F: 5'-TGCTATCCACCCTCAAACAGG-3'	286	(12)
	R: 5'-AACGTTGTAACCACCCCAAGA-3'		

## RESULTS

In this study, 162 non-duplicated *S. aureus* isolates were collected from four teaching hospitals in Isfahan (Table 2). Of 162 isolates, 48 (30%) and 114 (70%) were clinical isolates and nasal isolates respectively. In regards to demographic characteristics, 97 males (59.9%) and 65 females (40.1%), with the average age of 42 years (ranged 1–86 years), were among the subjects. Among 162 *S. aureus* isolates, 55 (34%) were MRSA (*mec*A positive) and 107 (66%) were methicillin-susceptible *S. aureus* (MSSA). Our findings revealed that 71 (43.8%) of *S. aureus* isolates were resistant to erythromycin. Furthermore, 87 isolates (51.2%) were susceptible to both erythromycin and clindamycin. Among 114 nasal carriage isolates, 48 (42.1%) were erythromycin-resistant and of these, 60.4% and 39.6% were cultured from patients and HCWs respectively. Moreover, out of 48 clinical *S. aureus* isolates, 23 (47.9%) were erythromycin-resistant.

**Table 2.** Distribution of phenotypic patterns based on different teaching hospitals

**N (%) of isolates**	**cMLS_B_ N=52 (%)**	**iMLS_B_ N=10 (%)**	**MS_B_ N=9 (%)**	**LS_A_ N=4 (%)**	**NEG N=87 (%)**	**Total N=162 (%)**
Alzahra	29 (56)	9 (90)	2 (22)	0	37 (43)	77 (48)
Shariati	6 (12)	0	4 (44)	3 (75)	32 (37)	45 (28)
Imam kazem	13 (25)	1 (10)	2 (22)	1 (25)	12 (14)	29 (18)
Shahidchamran	4 (8)	0	1 (11)	0	6 (7)	11 (7)

The most common erythromycin-resistant pheno-types were cMLS _B_, iMSL _B_, MS _B_, and LS _A_, respectively (Table 3). The majority of MRSA isolates (69%) showed cMLS _B_ phenotype. In addition, 7.2%, 5.4% and 1.8% of MRSA isolates had iMLS _B_, MS _B_ and LS _A_ phenotype, respectively.

**Table 3.** Distribution of studied genes (*erm*A, *erm*C, *msr*A) based on resistance phenotypes of *S. aureus* isolates

**N (%) of isolates**	***erm*A**	***erm*C**	***msr*A**	***erm*A** ***erm*C**	***erm*C** ***msr*A**	***erm*A** ***msr*A**	***erm*A** ***erm*C** ***msr*A**	**Negative PCR (for all 3 genes)**	**Total N=162**
cMLS_B_	7 (13)	10 (19)	1 (1)	9 (17)	8 (15)	1 (1)	10 (19)	6 (12)	52 (32)
iMLS_B_	1 (10)	5 (50)	1 (10)	0	2 (20)	0	0	1 (10)	10 (6)
MS_B_	0	3 (33)	0	1 (11)	2 (22)	0	1 (11)	2 (20)	9 (6)
LS_A_	0	1 (25)	0	0	0	0	0	3 (75)	4 (2)
NEG	2 (2)	3 (3)	1 (1)	1(1)	1 (1)	0	0	79 (92)	87 (54)

The most common erythromycin-resistant genes in *S. aureus* isolates were *erm*C 35.2% (57/162), *erm*A 20.4% (33/162) and *msr*A 17.3% (28/162). Of these, 34 (60%) out of 57 *erm*C positive isolates, 21 (64%) out of 33 *erm*A positive isolates and 24 (86%) out of 28 *msr*A positive isolates were MRSA (Table 4). Among 87 isolates with negative erythromycin-resistant phenotypes, 8 isolates (9%) carried at least one of the three genes *erm*C, *erm*A and *msr*A. Meanwhile, nine erythromycin-resistant, did not carry any of *erm* or *msr*A.

**Table 4.** Distribution of *erm*A, *erm*C and *msr*A genes among *S. aureus* isolates in clinical and nasal samples

**Genotype**	**MRSA**		**MSSA**		**Total N= (162)**
	
	**Nasal isolates N=35**	**Clinical isolates n=20**	**Nasal isolates N=79**	**Clinical isolates N=28**	
***erm*A**	3 (9)	2 (10)	4 (5)	1 (4)	10 (6)
***erm*C**	7 (20)	2 (10)	6 (8)	7 (25)	22 (14)
***msr*A**	1 (3)	1 (5)	1 (1)	0	3 (2)
***erm*A**	5 (14)	1 (5)	2 (3)	3 (11)	11 (7)
***erm*C**					
***erm*C**	7 (20)	3 (15)	2 (3)	1 (4)	13 (8)
***msr*A**					
***erm*A**	1 (3)	0	0	0	1 (1)
***msr*A**					
***erm*A**	6 (17)	5 (25)	0	0	11 (7)
***erm*C**					
***msr*A**					

## DISCUSSION

In this study, the rate of resistance to erythromycin was 43.8%, which is in agreement with reports from Kerman, Tabriz, Ahvaz (17–19), and lower than three studies from Tehran (2, 5, 20). The overall data show that the most prevalent phenotype was cMLS _B_ (32%), followed by iMLS _B_ (6%), MS_B_ (6%) and LS _A_ (2%), which is almost similar to the previous studies carried out in Isfahan, Tabriz, Kerman (8, 17, 18), and reprots from Brazil and India (13, 21). Our study also showed, there was some variation of the patterns of MLS resistance in different teaching hospitals. This may be due to differences in drug prescription, and consumption rates of macrolides and lincosamides in these hospitals. Our study showed that cMLS _B_ phenotype was dominant among the MRSA isolates. The high frequency of the cMLS _B_ in MRSA strains emphasizes on the importance of local surveillance in producing pertinent local resistance data, for appropriate empiric therapy. In addition, in our study the proportion of cMLS _B_ among MRSA isolates is higher than the result obtained from Kerman, and slightly lower, compared to another study from Japan (17, 22). The findings of our study demonstrate that *erm*C (35.2%) is predominant, relative to the *erm*A (20.4%) and *msr*A (17.3%). In addition, the rate of prevalence of *erm*C, *erm*A and *msr*A, among erythromycin-resistant isolates were 71.8%, 42.2%, 36.6%, respectively. Prevalence of these genes in reports from different cities and countries were variable. For example, some Iranian studies have shown that *erm*A was responsible for the majority of resistance to erythromycin (2, 5, 19), while in the others *erm*C was the most common (8, 18, 23, 24). Differences in results were also shown in other countries. In a study conducted by Duran et al. in Turkey, the prevalence of *erm*A and *erm*C were reported as 52% and 28%, respectively (25). Schmitz et al. analyzed *S. aureus* isolates from 24 European university hospitals, and reported that *erm*A (67%) was more prevalent than *erm*C (23%) (26). Furthermore, similar results were reported from Korea (27). Consistent with the present study, *erm*C was predominant, compared to results from some studies in Brazil, Turkey, and Greece (10, 28, 29). However, some points must be highlighted, which were emerging from *erm*C, particularly in the MRSA strains. The results show that the *erm*C can be easily transferred by plasmids to other species, that may be due to the local antibiotic policies. A notable finding of the present study was the high prevalence of *msr*A among *S. aureus* isolates (17.3%) and erythromycin-resistant isolates (36.6%) in Isfahan. Few studies reported, a high prevalence of this gene in Iran (20, 21). In our study, the rate of *msr*A in MRSA isolates was 43.6% which is almost similar to results obtained by Nezhad et al. (20). In contrast to our finding, Goudarzi et al. found that among 51 *S. aureus* isolates, only 3.9% had *msr*A(24) and three studies in Iran did not detect any *msr*A (2, 18, 19). Moreover, in this study, most of *msr*A carried by MRSA isolates and a significant association was observed between the presence of *mec*A and *msr*A. Data from these studies suggest that MRSA strains are successful in the acquisition and spread of *msr*A among *S. aureus* isolates.

Another notable result of our study, was the high rate of co-existence of *erm*A, *erm*C and *msr*A, which indicates that the combination of resistance mechanisms in Isfahan is expanding. In the present study, the co-existence of *erm*A+*erm*C+*msr*A was detected only in MRSA isolates. Moreover, 10 out of 13 isolates with *erm*C+*msr*A genotype and one isolate with *erm*A+*msr*A genotype were MRSA. It is well documented that cMLS _B_ phenotype characterized by the presence of a great number of gene combinations, which is consistent with our findings (30). These results suggested that *mec*A plays an important role, in the development of different mechanisms of resistance in isolates of Isfahan.

In conclusion, our results demonstrated that cMLS _B_ and *erm*C are the most frequent phenotypes and genes, respectively, in the hospitals of Isfahan and distribution of phenotypes is variable in different hospitals. This report was the first study to demonstrate the high frequency of coexistence of *erm*A+*erm*C+*msr*A, in Isfahan. According to our results, we recommend to perform routine D-test in teaching hospitals to conduct local periodic study in all hospitals.
